# Refined Mapping of Endothelial Cells, Brain Pericytes and Astrocytes Proteomes via Subcellular Fractionation and DDA Mass Spectrometry

**DOI:** 10.3390/biology15191724

**Published:** 2026-10-01

**Authors:** Océane Dusailly, Camille Menaceur Vandenbroucke, Johan Hachani, Sophie Duban-Deweer, Fumitaka Shimizu, Takashi Kanda, Yannis Karamanos, Fabien Gosselet, Laurence Fenart, Julien Saint-Pol

**Affiliations:** 1 Univ. Artois, UR 2465, Blood-Brain Barrier Laboratory (LBHE), F-62300 Lens, Francecamen@mbg.au.dk (C.M.V.); johan.hachani@univ-artois.fr (J.H.); sophie.duban@univ-artois.fr (S.D.-D.); yannis.karamanos@univ-artois.fr (Y.K.); fabien.gosselet@univ-artois.fr (F.G.); laurence.tilloy@univ-artois.fr (L.F.); 2 Department of Neurology and Clinical Neuroscience, Graduate School of Medicine, Yamaguchi University, Ube, Japan; fshimizu@yamaguchi-u.ac.jp; 3 Department of Neurology, Neuromuscular Center Yoshimizu Hospital, Shimonoseki, Japan; tkanda@yamaguchi-u.ac.jp

**Keywords:** data-dependent analysis, endothelial cells, brain pericytes, astrocytes

## Abstract

Proteomic profiling of brain microvascular cells, i.e., endothelial cells, brain pericytes, and glial cells, is essential for understanding their distinct roles in the neurovascular unit. In this study, we present a comparative data-dependent acquisition (DDA) analysis of endothelial cells (ECs), brain pericytes (BPs), and astrocytes (ACs), using both whole-cell lysates (WCL) and subcellular fractionation. Analysis of the WCL identified 2000–2300 proteins per cell type, revealing distinct biological signatures consistent with the known function of each cell type. Compared to the standard WCL protocol, subcellular fractionation significantly increased the depth of the proteome (by up to 93%), particularly for membrane and insoluble proteins. Functional enrichment analysis revealed sharper biological associations: angiogenesis in ECs, cell cycle regulation in BPs, and lipid metabolism in ACs. However, the results also revealed that the sampling method impacts the interpretation of cell-type specificity. For example, a disintegrin and metalloprotease 10 (ADAM10) appeared EC-specific in WCL data but was broadly detected after fractionation. Likewise, Glial Fibrillary Acidic Protein (GFAP) was unexpectedly identified in ECs, reflecting its transient expression in immature cells. To overcome such biases, we generated an integrative reference proteome for each cell type by combining data from different sampling strategies. These include 3605 proteins for ECs, 3378 for BPs, and 3976 for ACs, with consistent representation of membrane-associated sub-proteomes. This study highlights the importance of integrating sampling methods and considering cell differentiation status when interpreting proteomic data. It also provides valuable reference datasets for future neurovascular research.

## Simple Summary

This study presents an integrative proteomic analysis of endothelial cells, pericytes, and astrocytes—the three main cell types orchestrating vascular and cerebral metabolism and functions—using whole-cell lysates and subcellular fractionation combined with data-dependent acquisition mass spectrometry. Subcellular fractionation enhanced proteome coverage by up to 93%, especially for membrane-associated and insoluble proteins, revealing sharper cell-type-specific functional signatures. However, sampling strategy and cell differentiation status were shown to influence protein detection and interpretation of specificity. By merging data across approaches, we generated reference proteomes for each cell type, offering valuable resources for neurovascular research and biomarker discovery.

## 1. Introduction

Endothelial cells (ECs), pericytes (BPs), and astrocytes (ACs) represent three major cellular populations that orchestrate vascular and metabolic functions in the central nervous system. ECs line cerebral microvessels and regulate the selective exchange of molecules between the circulation and neural tissue, ensuring tight control over nutrient delivery and waste clearance [1,2]. BPs, embedded within the vascular basement membrane, provide structural support, stabilize nascent vessels, and regulate angiogenesis and vascular remodeling [3,4]. They also engage in extensive crosstalk with ECs, modulating their phenotype and barrier properties through soluble factors and direct contact-dependent signaling [5]. ACs, with their perivascular endfeet, maintain ionic and metabolic homeostasis, recycle neurotransmitters, and provide essential metabolic substrates to neurons [6,7].

Mass spectrometry (MS)-based proteomics has emerged as a key strategy for global protein profiling in complex tissues and isolated cell populations. Among the various MS acquisition methods, data-dependent acquisition (DDA) remains widely used due to its robustness and flexibility [8]. However, the analysis of primary brain cell types using proteomics is still challenged by the intrinsic heterogeneity of samples due to the isolation difficulties, differences in protein solubility, and the subcellular distribution of proteins. These factors can significantly impact protein recovery, identification, and specificity of the results.

Traditionally, whole-cell lysate (WCL) preparation has served as a standard approach in proteomics due to its simplicity and broad protein coverage. However, as we observed in a previous study on BPs [9], WCL methods often disproportionately favor highly abundant cytosolic proteins, which can mask less abundant or poorly soluble proteins, such as membrane-bound or cytoskeletal proteins [10,11,12]. This bias reduces the sensitivity to key proteins involved in signal transduction, transport, and structural organization-critical features in neurovascular cell types.

To overcome these limitations, subcellular fractionation protocols have been used to separate the cellular content into distinct biochemical compartments, namely cytoplasmic, membrane, nuclear, and insoluble fractions [13,14,15]. These methods have been shown to enhance the depth of the proteome, particularly for membrane proteins, and to facilitate the functional dissection of sub-proteomes in both data-dependent and data-independent workflows [16,17,18]. Moreover, fractionation may increase the accessibility of proteins that are difficult to digest for enzymatic cleavage, further improving identification efficiency [19].

While proteome fractionation enhances coverage, it can also introduce complexity in data interpretation, particularly when assessing cell-type-specific protein expression. The sampling strategy may directly affect whether a protein appears to be enriched or restricted to a given cell type. As demonstrated in studies of a disintegrin and metalloprotease 10 (ADAM10), a widely expressed transmembrane metalloprotease, its apparent cell-type specificity can vary depending on whether proteins are sampled from WCL or enriched membrane fractions [20,21]. Similarly, markers such as Glial Fibrillary Acidic Protein (GFAP), typically associated with mature astrocytes, may be transiently expressed in immature or undifferentiated endothelial cells during the early stages of differentiation, confounding cell-type attribution [22,23].

Therefore, a rigorous definition of cell-type proteomes must address some critical parameters: the protein sampling strategy (e.g., WCL vs. subcellular fractions), the composition of cell culture media, and the differentiation status of the cells studied. These factors are particularly relevant when defining reliable cell-type markers or investigating proteins involved in brain development and pathology.

In the present study, a comprehensive proteomic analysis of ECs, BPs, and ACs using both WCL and subcellular fractionation-based approaches was performed with three main goals: (i) to evaluate the influence of different sampling strategies on global proteome coverage and detection of sub-proteomes; (ii) to assess the impact of sampling methods on the interpretation of cell-type-specific protein expression; and (iii) to compile robust reference proteomes for each brain cell type based on our findings. We further emphasize how sampling bias, cellular context, and the composition of cell culture media can mislead interpretations of protein specificity and cell differentiation evaluation.

By providing an integrative view of the proteomes of ECs, BPS, and ACs across sampling modalities, the present study contributes both methodologically and biologically to the advancement of proteomic studies in the central nervous system.

## 2. Materials and Methods

### 2.1. Reagents and Chemicals

Accutase was obtained from StemCell Technologies (Vancouver, BC, Canada). Tris–HCl, guanidine hydrochloride (GuHCl), ethylenediaminetetraacetic acid (EDTA), dithiothreitol (DTT), iodoacetamide, ammonium bicarbonate (NH_4_HCO_3_), formic acid, and acetonitrile (ACN) were purchased from Sigma-Aldrich (St. Louis, MO, USA). Quick Start Bradford dye reagent was acquired from Bio-Rad (Hercules, CA, USA). Mass spectrometry-grade Trypsin Platinum was obtained from Promega (Madison, WI, USA). HyperSep SpinTip Microscale C18 columns and the Quantitative Colorimetric Peptide Assay were purchased from Thermo Fisher Scientific (Hampton, NH, USA). The Total RNA/Protein Isolation Kit and QProteome Cell Compartment Kit were obtained from Qiagen (Hilden, Germany). PepCalMix LC-MS calibration solution was purchased from AB Sciex (Foster City, CA, USA).

### 2.2. Cells and Cell Culture

All cell cultures were regularly screened for mycoplasma contamination using the MycoAlert PLUS Mycoplasma Detection Kit (Lonza, Basel, Switzerland, catalog no. LT07-710), and only cultures that tested negative were used in the experiments.

#### 2.2.1. Human Endothelial Cells (ECs)

Human hematopoietic stem cells were isolated from umbilical cord blood, as previously described [24,25,26], and used at passage 6 for experiments. After 15 to 20 days, CD34+ cells were induced to differentiate into endothelial cells (ECs) and subsequently cultured in 100 mm gelatin matrix-coated Petri dishes (0.2%). The culture medium consisted of Endothelial Cell Medium (PromoCell endothelial cell basal medium MV2 (Basal 514M172) with Promocell supplement mix endothelial cell growth medium MV2 (Mix 512M269)). The cells were maintained at 37 °C in a humidified incubator with 5% CO_2_.

#### 2.2.2. Human Brain Pericytes (BPs)

Human brain pericytes (BPs) were kindly provided by Professor Takashi Kanda’s team (Department of Neurology and Clinical Neuroscience, Yamaguchi University Graduate School of Medicine, Ube, Japan). In brief, following their isolation from brain tissue, the pericytes were immortalized through transfection with retroviral vectors encoding the temperature-sensitive SV40 T antigen (tsA58) and human telomerase (hTERT) [25,27]. BPs are used at passage 10 for experiments. Upon thawing, 6.25 × 10^5^ hBPs, used between passages 15 and 25, were seeded on gelatin-coated (Sigma-Aldrich, Saint Louis, MS, USA, G-2500) 100 mm Petri dishes and cultured in Dulbecco’s Modified Eagle’s Medium (DMEM; Life Technologies) supplemented with 4.5 g/L glucose, 10% fetal calf serum (FCS, Life Technologies, Waltham, MA, USA), 2 mM L-glutamine, and 1% antibiotics (penicillin and streptomycin).

#### 2.2.3. Human Astrocytes (ACs)

Culture flasks (75 cm^2^, Sarstedt #83.3911.002) were pre-coated with Poly-L-lysine (PLL) at a concentration of 2 µg/cm^2^ in sterile water. Human cerebral cortex astrocytes (HA-bc, ScienCell #1800, Carlsbad, CA, USA, 10^6^ cells/vial, passage 5) were thawed and cultured in a specialized astrocyte medium (AM), consisting of AM base medium (ScienCell #1801), 2% heat-inactivated fetal bovine serum (FBS; Sigma #F7524, lot BCBR5156V), 1% Astrocyte Growth Supplement (AGS; ScienCell #1852), and 1% penicillin/streptomycin (Gibco #15140-122) and plated into the previously PLL-coated flask [25,28]. Cells were incubated at 37 °C in a controlled environment with 5% CO_2_ and regulated humidity. After 16 h, the culture medium was replaced to remove residual dimethyl sulfoxide (DMSO) and any unattached cells. Thereafter, the medium was refreshed every three days to maintain optimal cell growth and viability.

### 2.3. Sample Preparation for Label-Free Mass Spectrometry (MS)

Protein extracts from the three cell types were prepared from four independent batches per cell type, which were treated with Accutase, and the detached cells were pelleted by centrifugation at 300× *g* for 5 min at 4 °C. The cells were then rinsed three times with phosphate-buffered saline (PBS, pH 7.4) and centrifuged for 5 min at 300× *g* at 4 °C, and the final pellets were stored at −80 °C until preparation for MS analysis. Two batches correspond to the whole-cell lysate (WCL) conditions. The two other batches were processed using the QProteome Cell Compartment Kit (Qiagen, #37502) using the manufacturer’s protocol to provide cytoplasmic protein fractions (F1), membrane protein fractions (F2), nuclear protein fractions (F3), and insoluble protein fractions (F4). The fraction pellets were stored at −80 °C until preparation for MS analysis.

Total proteins from cells (WCL) and fractions (F1, F2, F3 and F4) were extracted using a 50 mM Tris-HCl buffer (pH 7.5) containing 6 M guanidine hydrochloride (GuHCl) for 5 min at 4 °C. The samples were purified from cell debris and contaminating DNA with the Total RNA Protein Isolation kit following the manufacturer’s recommendations. Protein concentration was determined using the Quick Start Bradford dye reagent (Bio-Rad). Then, the sulfhydryl groups of 100 µg of protein were reduced with 25 mM dithiothreitol (DTT) *w*/*v* in 25 mM ammonium bicarbonate (NH_4_HCO_3_) for 20 min at 56 °C, carbamidomethylated with iodoacetamide used in a 2.5-fold excess (*w*/*w*) in the dark (20 min, RT), and purified by ice-cold 80% acetone precipitation, overnight at −20 °C. The precipitate was recovered by centrifugation at 14.000× *g* for 10 min at 4 °C, and the proteins were enzymatically digested overnight using Trypsin Platinum, Mass Spectrometry Grade, with an enzyme/substrate ratio of 1:10 at 37 °C in 25 mM NH_4_HCO_3_ according to the supplier’s protocol. The enzymatic reaction was stopped in 0.2% (*v*/*v*) formic acid in water. The peptides were extracted using the HyperSep SpinTip Microscale C18, and the peptide concentration was estimated using the Quantitative Colorimetric Peptide Assay kit. The samples were prepared with a final concentration of 1 µg/µL in a solution of 2% acetonitrile (ACN) and 0.1% (*v*/*v*) formic acid in water before MS analysis.

### 2.4. Data-Dependent Analysis

Data-dependent acquisition by mass spectrometry (DDA-MS) is a proteomic approach in which digested peptides are analyzed by LC-MS/MS. Peptide signals that were detected in a full-scan mass spectrum with predefined MS parameters are selected for fragmentation, and then their product (MS/MS) mass spectra are matched to spectra in a protein database (UniProtKB, https://www.uniprot.org, accessed on 1 June 2026).

To generate the protein libraries, 2 µg samples of protein from each condition were analyzed using an LC-MS/MS system, an AB Sciex TripleTOF 5600+ mass spectrometer (AB Sciex, Foster City, CA, USA) coupled to an Ekspert nanoLC 400 System. Each sample was injected 5 times (N = 2, *n* = 10), and a PepCalMix LC-MS solution (AB Sciex) was used for calibration after each injection, allowing for maintenance of a mean mass error below 10 ppm.

Briefly, two μg of peptides from each sample were loaded on a trap column (Luna Omega 5 µm Polar, C18, 100 Å, Micro Trap 20 × 0.5 mm, Phenomenex, Torrance, CA, USA) with a rate of 10 µL/min for 3 min and then separated using an Eksigent Chrom XP-C18 HPLC reverse-phase column (0.3 × 150 mm, 120 Å, particle size 3 µm, Sciex, Framingham, MA, USA) using a mobile phase gradient composed of 0.1% (*v*/*v*) formic acid in 2% ACN 98% water (phase A) and 0.1% (*v*/*v*) formic acid in 98% ACN 2% water (phase B) with a flow rate of 5 µL/min. The gradient was as follows: 2 to 8% solvent B in A (from 0 to 5 min), 8 to 35% B (5 to 90 min), 35 to 40% B (90 to 100 min), 40 to 90% B (100 to 102 min), 90% B (102 to 107 min), 90 to 2% B (107 to 109 min) and finally 2% solvent B in A (109 to 140 min). The total runtime was 140 min, including a mobile phase equilibration. The autosampler and column temperatures were maintained at 8 °C and 35 °C, respectively.

The eluted peptides were analyzed directly for MS data acquisition using an AB Sciex TripleTOF 5600+ system, which was then operated in positive ion mode by electrospray ionization with the DuoSpray^®^ (Sciex) and Turbo V^®^ (Sciex) ionization sources and controlled by the Analyst software (version 1.7.1). The Ionization parameters were as follows: ISVF = 5500, GS1 = 15, GS2 = 15, CUR = 25, TEM = 150. MS spectra were acquired in high-sensitivity (HS) mode for 250 ms from 400 to 1250 *m*/*z,* and MS/MS scans from 100 to 1500 *m*/*z* (60 ms accumulation time, 20 ppm mass tolerance, rolling collision energy). The generation of protein libraries was performed using the ProteinPilot software (AB Sciex, version 5.0.2) with a UniProtKB human-filtered proteome database. Cysteine carboxymethylation and methionine oxidation were specified as variable modifications, as were two trypsin miscleavages. The threshold for detecting proteins was set to 10% (unused ProtScore > 0.05). The false discovery rate (FDR) was used to generate a spectral library and was set to 1% for both peptides and proteins.

### 2.5. Meta-Analysis of MS Data

The data generated by MS were analyzed using JVenn [29] (https://jvenn.toulouse.inrae.fr/app/index.html, accessed on 17 May 2026) to display common and specific proteins, the Panther 18.0 [30,31] (https://archive.pantherdb.org, accessed on 2 June 2026) and STRING 12.0 [32] (https://string-db.org, accessed on 25 May 2026) databases to assess the functions of the identified proteins and their interaction networks, respectively. Gene Ontology (GO) analytical data were obtained through MetaScape [33] (https://metascape.org/gp/index.html#/main/step1, accessed on 16 May 2026). All data were generated exclusively for proteins that were significantly identified with a minimum of two peptides. GO:001620 and GO:0005886 protein databases, corresponding to membrane proteins and plasma membrane proteins, respectively, were obtained from UniProt [34] (https://www.uniprot.org, accessed on 1 June 2026).

## 3. Results and Discussion

### 3.1. First Comprehensive Proteomic Profiling of Endothelial Cells, Brain Pericytes, and Astrocytes from Whole-Cell Lysates

The proteomic analysis of proteins expressed in endothelial cells (ECs), brain pericytes (BPs), and astrocytes (ACs) using whole-cell lysate (WCL) samples was carried out as described in the Materials and Methods section. WCLs were prepared from pelleted cells and enzymatically digested with trypsin. This classical and widely used sample preparation method enables rapid data-dependent acquisition (DDA) and efficient identification of whole-cell proteomes. Only proteins with *p*-values < 0.01 are considered significant.

As shown in Figure 1A, the analysis identified 2145 proteins in ECs, 2328 in BPs, and 2016 in ACs (Table S1). Among these, 1292 proteins were shared across all three cell types, while 330 proteins were common to ECs and BPs, 363 to BPs and ACs, and 105 to ECs and ACs. The Venn diagram also highlighted cell type-specific proteins: 418 unique to ECs, 343 to BPs, and 256 to ACs. The top-20 Gene Ontology (GO) pathways enriched among these cell type-specific proteins are shown in Figure 1B–D. In ECs (Figure 1B), the specific proteins are primarily associated with mitochondrial function, vascular development, and hemostasis. In BPs (Figure 1C), the predominant functions are related to cell development, mitosis, and proliferation. For ACs (Figure 1D), the specific proteins are linked to vesicle-mediated transport and endocytosis, as well as sterol-regulated pathways involving Sterol Regulatory Element-Binding Proteins (SREBPs). Overall, this WCL-based proteomic analysis successfully identified distinct protein signatures that align with the expected biological functions of ECs, BPs, and ACs.

### 3.2. Cell Lysate Fractionation Enhances Protein Identification and Provides a More Focused Functional Characterization of Cell-Type-Specific Proteins

The second step was to compare the WCL proteomes of ECs, BPs, and ACs with those obtained after cell fractionation, which separated the lysates into four distinct subcellular fractions: cytoplasmic proteins (F1), membrane proteins (F2), nuclear proteins (F3), and insoluble proteins (F4). Previous studies have demonstrated that such fractionation approaches enhance protein identification [14,17].

Figure 2A summarizes the number of proteins identified in each fraction for ECs, BPs, and ACs (Table S2, Table S3, and Table S4, respectively). Compared to ECs and BPs, which showed a similar distribution of identified proteins across fractions, ACs yielded a greater number of cytoplasmic and membrane proteins but fewer insoluble proteins. Although the cumulative (naïve) sum of proteins identified across fractions is provided for reference, it does not reflect the true number of distinct proteins identified, since many proteins are present in multiple fractions—i.e., two, three, or four fractions—because of cross-contamination between fractions. To address this, a list of non-redundant proteins identified across all four fractions (NRP Fr.) was compiled to define the global proteome for each cell type. Compared to the WCL proteomes, the NRP Fr. approach led to a significant increase in protein identification: +56.7% in ECs, +39.3% in BPs, and +92.8% in ACs. This enrichment can be attributed to the limitations of WCL sampling, where abundant cytoplasmic proteins may hinder the detection of less abundant proteins and compromise the digestion of insoluble or fibrillar proteins, such as those associated with the cytoskeleton [10,11,12].

As for the WCL datasets, Venn diagram analysis was used with the NRP Fr. proteomes to identify cell-type-specific proteins (Figure 2B). Interestingly, the number of EC- and BP-specific proteins decreased in the NRP Fr. datasets compared to WCL (282 vs. 418 for ECs and 189 vs. 343 for BPs), while the number of AC-specific proteins increased substantially (838 in NRP Fr. vs. 256 in WCL). This shift can be partially explained by the increased number of shared proteins identified across all three cell types in the NRP Fr. dataset (2548 vs. 1292 in WCL).

The top-20 Gene Ontology (GO) pathways associated with cell-type-specific proteins in the NRP Fr. Proteomes were also examined. Unlike the WCL data, which highlighted multiple dominant functions per cell type, the NRP Fr. analysis revealed one predominant biological function per cell type: angiogenesis in ECs (Figure 2C), mitotic cell cycle regulation in BPs (Figure 2D), and lipid metabolism in ACs (Figure 2E).

In conclusion, subcellular fractionation not only enhances protein identification but also enables a more refined functional analysis of cell-type-specific proteins, offering clearer insights into cell-specific biological processes compared to conventional WCL-based proteomics.

### 3.3. The Comprehensive Use of All Subcellular Fractions Not Only Maximizes Protein Coverage but Also Mitigates the Protein Loss and Contamination Issues Inherent to Individual Fraction Analysis

Subcellular fractionation protocols are commonly used to enrich specific subproteomes and enable rapid identification of proteins of interest in cells and tissues [13,15,35,36,37]. With this goal in mind, we focused on the membrane protein fraction (F2) from endothelial cells (ECs) and compared the proteins identified in this fraction with those found in the WCL and NRP Fr. proteomes (Figure 3).

The number of membrane proteins identified in the ECs, WCL, and F2 datasets using the Gene Ontology term GO:001620, which refers to membrane-associated proteins (Figure 3A), was assessed. The F2 fraction enabled the identification of 222 additional membrane proteins not detected in the WCL sample. However, 195 membrane proteins identified in WCL were absent from the F2 fraction. This indicates that while subcellular fractionation enhances enrichment, it may also result in the loss of certain membrane proteins when compared to WCL.

In a second step, using the same GO term, the ECs F2 fraction was compared to the NRP Fr. proteome (Figure 3B). As expected, all proteins detected in the F2 fraction were also present in the NRP Fr. dataset. However, 193 membrane proteins identified in the NRP Fr. proteome were absent from the F2 fraction. This finding highlights a known limitation of fractionation protocols: the potential protein loss due to contamination across fractions or incomplete recovery from the target compartment.

In order to investigate the fate of these 193 “missing” membrane proteins, their distribution across the F1 (cytoplasm), F3 (nuclear), and F4 (insoluble) fractions (Figure 3C) was examined. Most of these proteins were detected in the F1 fraction, suggesting they may be glycosylphosphatidylinositol (GPI)-anchored or peripheral membrane proteins that associate transiently with membranes and are therefore recovered in the cytoplasmic fraction. Similar results were observed for BPs (Figure S1) and ACs (Figure S2), and are summarized in Table 1, which details the distribution of these “missing” F2 membrane proteins across F1, F3, and F4.

These findings emphasize the importance of caution when interpreting data from subcellular fractionation protocols. While such approaches can enrich specific subcellular compartments, they may fail to include certain proteins of interest. Moreover, as shown for the F2 fraction, some membrane-associated proteins may be detectable only in WCL samples, reinforcing the complementary nature of these sampling strategies. The intracellular fate, processing, and trafficking of proteins should also be considered when determining their predominant subcellular distribution. For example, a transmembrane protein may undergo proteolytic cleavage, releasing a soluble intracellular fragment that can function as a transcription factor. Consequently, the protein may be detected in the F1, F2, and/or F3 fractions, depending on the peptide sequences identified by MS. This observation highlights the importance of examining all fractions when investigating a given protein, rather than restricting the analysis to its expected or primary fraction.

### 3.4. NRP Fr. Proteomes Yield More Membrane and Plasma Membrane Protein Candidates than WCL Proteomes, While Maintaining Similar Proportions

As previously shown, subcellular fractionation improves the identification of membrane proteins compared to the WCL condition (Section 3.3). To further investigate this, we assessed the proportion of plasma membrane (PM) proteins identified under each sampling strategy to determine whether fractionation affects the relative abundance of PM proteins among the total membrane protein pool (Figure 4). Membrane proteins and PM proteins of the WCL and NRP Fr. proteomes of ECs, BPs, and ACs were identified using the Gene Ontology (GO) terms GO:001620 and GO:0005886, respectively.

Figure 4A illustrates the number of membrane proteins identified in ECs, BPs, and ACs under WCL (solid bars) and NRP Fr. (striped bars) conditions, as well as the proportion of PM proteins within each sample set (dark solid and striped bars). We demonstrate that the total number of identified membrane proteins is increased by fractionation compared to WCL, with increases of 27.2%, 31%, and 112% observed for ECs, BPs, and ACs, respectively. A similar trend is evident for the plasma membrane sub-proteome, with increases of 30.4%, 23.9%, and 88.5% for ECs, BPs, and ACs, respectively. These results support the advantage of cellular fractionation for optimizing the identification of proteins from specific sub-proteomes-here, the membrane sub-proteome and its various components. All identification data are summarized in Figure 4B.

However, the proportion of membrane or PM proteins identified under each sampling condition remains largely consistent. For instance, in ECs, membrane proteins represent 37.8% of the total proteome under WCL conditions, compared to 30.6% in the NRP fraction, while the proportion of PM proteins remains close to 10% in both sampling conditions. A similar trend is observed for BPs and ACs (Figure 4B). Therefore, while cellular fractionation facilitates the identification of proteins from various sub-proteomes-such as the membrane sub-proteome-it does not selectively enrich for membrane proteins. Instead, it results in a global enrichment across all sub-proteomes.

### 3.5. Protein Sampling Method for DDA Analyses Can Lead to Misinterpretations of Cell-Type Specificity

A major advantage of mass spectrometry-based studies across different cell types is the ability to rapidly identify and highlight proteins that are specifically expressed by each cell type, referred to as cell-type specificity. However, depending on the sampling strategy employed, misinterpretations or aberrations in specificity may arise. To illustrate this point, the example of ADAM10 was selected.

ADAM10, also known as alpha-secretase, is a pleiotropically expressed catalytic transmembrane protein that cleaves 60 to 100 distinct substrates mainly at the cell PM side, including CD44, Notch, and the amyloid precursor protein (APP) [20,21,38] (Figure S3A). It has been shown that the activity and subcellular localization of ADAM10 are regulated by a subset of tetraspanins known as TspanC8. These are characterized by eight conserved cysteine residues in their large extracellular loop [21,39]. This group includes Tspan5, Tspan10, Tspan14, Tspan15, Tspan17, and Tspan33 [40]. These tetraspanins interact directly with ADAM10, suggesting a finely tuned regulatory mechanism dependent on the specific TspanC8 involved, the substrate being processed, and the cell type, reflecting the differential or cell-type-dependent expression of TspanC8 members [21,41,42,43,44,45]. Among these, Tspan14 is known to be specifically expressed, or at least enriched, in ECs [46,47,48,49].

With these considerations in mind and given that Tspan14 is specific to ECs, the potential cell-type specificity of ADAM10 expression in ECs was assessed. The approach previously described, intersecting EC-specific proteins identified in WCL (Figure 1) and NRP Fr. (Figure 2) with GO terms GO:001620 and GO:0005886 for membrane proteins and PM proteins, respectively (Figure S3B,C), was employed. PM proteins were then analyzed using the STRING database to generate protein–protein interaction networks [32] (Figure 5). Under WCL conditions (Figure 5A), we confirmed the presence of Tspan14 in the EC PM interactome and also identified ADAM10 in direct interaction with Tspan14. Based on these results, it can be concluded that ADAM10 displays a selective expression profile across ECs, BPs, and ACs, suggesting it is EC-specific. The same analysis was performed under NRP Fr. conditions (Figure 5B). While Tspan14 was still present in the EC PM interactome, ADAM10 was not detected, indicating that under this sampling condition, ADAM10 is not EC-specific. Thus, depending on the protein sampling strategy, conclusions regarding the cell-type specificity of ADAM10 differ.

Upon inspecting the raw DDA data for both WCL and NRP Fr., ADAM10 is indeed identified exclusively in ECs under WCL conditions, with peptide counts in BPs and ACs falling below the significance threshold (Figure S3D). In contrast, under NRP fractionation, ADAM10 is significantly detected in all three cell types, with 11 peptides in fraction F2 and two in F4 for ECs, four peptides in F2 for BPs, and 13 peptides in F2 for ACs (Figure S3D). This demonstrates that cellular fractionation enhances the detection of ADAM10 across all three cell types, thereby supporting the conclusion that ADAM10 is pleiotropically expressed [20,21,38]. Hence, the choice of protein sampling strategy can be critical in avoiding misinterpretations regarding the degree of expression of a protein of interest, or the cell-type specificity of a given protein.

### 3.6. Identifying Cell-Type Markers Requires Careful Consideration of Protein Sampling and of Cellular Differentiation Status

This point is illustrated using the example of Glial Fibrillary Acidic Protein (GFAP). As its name suggests, GFAP is a protein that is predominantly expressed in glial cells, particularly astrocytes [50,51,52]. GFAP is a fibrillar, insoluble protein, and as such, it is notoriously difficult to detect by DDA mass spectrometry. The proteomes of ECs, BPs, and ACs obtained both with WCL and NRP Fr. were compared (Figure 6). The focus first turns to the AC proteome in WCL samples. Given GFAP’s enrichment in astrocytes, particular attention was paid to AC-specific proteins. Under WCL conditions, GFAP expression was not detected in the AC proteome (Figure 6A), nor in proteins shared with ECs, with BPs, or with both.

In contrast, with NRP Fr. (Figure 6B), GFAP expression was surprisingly detected in the shared protein pool between ECs and ACs, specifically in the F4 fraction enriched for insoluble proteins (Figure 6C). This unexpected observation is nevertheless significant, as evidenced by the number of peptides identified for GFAP in ECs (14 peptides) and in ACs (8 peptides, Figure 6D). While seemingly aberrant, the shared detection of GFAP in ECs and ACs can be explained by considering (i) the absence of cross-contamination of samples since AC and EC samples have been processed independently for DDA, and (ii) the differentiation status of the cells analyzed. Indeed, GFAP expression would be considered artifactual or aberrant in mature, fully differentiated ECs-such as brain endothelial cells forming the blood–brain barrier (BBB). However, it has been shown that undifferentiated ECs can transiently express GFAP during early stages of differentiation, likely linked to cytoskeletal reorganization, with this expression diminishing as differentiation progresses [22,23].

Thus, to identify a reliable cell-type marker, two key parameters must be considered: (i) the protein sampling strategy, which can greatly enhance the detection of certain protein classes—such as insoluble proteins—in DDA, and (ii) the differentiation state of the cells being studied.

Another point requiring careful consideration is the composition of the culture medium, which can substantially influence astrocyte behavior, transcriptional identity, maturation, and reactivity, particularly in monoculture. Astrocytes maintained in serum-containing media frequently diverge from their in vivo phenotype, whereas defined serum-free conditions may better preserve mature and homeostatic characteristics; however, these effects depend on the species, cell source, isolation procedure, duration of exposure, and medium formulation [53,54,55,56]. We therefore examined the expression of canonical astrocytic markers to assess the potential influence of fetal bovine serum (FBS) supplementation. As shown in Table S5, astrocytes (ACs) displayed detectable expression of glial fibrillary acidic protein (GFAP), glutamine synthetase (GS; encoded by GLUL), connexin 43 (Cx43; GJA1), clusterin (CLU), CD44, and vimentin (VIM). Conversely, no detectable expression was observed for several other commonly used astrocytic markers, including aquaporin 4 (AQP4), excitatory amino acid transporter 1 (EAAT1; SLC1A3), excitatory amino acid transporter 2 (EAAT2; SLC1A2), S100 calcium-binding protein B (S100B), and cytosolic 10-formyltetrahydrofolate dehydrogenase (ALDH1L1). ALDH1L1, AQP4, and the astrocytic glutamate transporters are informative components of mature or homeostatic astrocyte profiles, whereas GFAP, VIM, and CD44 may also be expressed or upregulated in reactive states [53,54,56,57,58]. Taken together, the marker profile observed under FBS-supplemented conditions is consistent with an incompletely mature, serum-associated reactive-like phenotype rather than a fully differentiated homeostatic state. Nevertheless, because no individual marker or fixed marker panel unequivocally defines astrocyte identity or reactivity, this interpretation should ideally be supported by morphological assessment, quantitative expression analyses, functional assays, and direct comparison with a defined serum-free condition [58]. Culture–medium composition should therefore be regarded as a critical experimental variable when identifying and interpreting cell-specific marker profiles. Moreover, some markers are present in WCL, and some are only identified after fractionation, such as for CLU, underscoring the complementarity of sampling methods to optimize the identification of cell markers.

### 3.7. Defining the Most Comprehensive and Accurate Reference Cell Proteome Requires Integrating Protein Identifications from Multiple Sampling Strategies

Taking into account the previous considerations regarding (i) the inherent incompleteness of proteomes associated with each sampling method, (ii) the challenges in identifying insoluble proteins, and (iii) the risk of misinterpreting cell-type-specific expression data, we generated a pooled dataset of DDA-based protein identifications obtained from both WCL and NRP Fr. for ECs, BPs, and ACs that were referred to as reference proteomes (Table 2 and Table S6).

The reference proteome established for each cell type considers the specific contributions of each sampling method, referred to as “specific proteins per method” in Table 2, resulting in the identification of 3605 total proteins for ECs, 3378 proteins for BPs, and 3976 proteins for ACs. The membrane sub-proteome includes 1178 proteins, of which 419 are PM proteins, for ECs; 1019 proteins, including 364 PM proteins, for BPs; and 1431 proteins, including 489 PM proteins, for ACs. In line with the analysis presented in Section 3.4, membrane proteins represent approximately 33% of the total proteome, and PM proteins account for around 11.5%, which is consistent with the proportions observed in the WCL and NRP fractionation-derived proteomes.

We then analyzed the cell-type-specific proteomes (Figure 7A) and identified 334 proteins unique to ECs, 203 proteins specific to BPs, and 716 proteins specific to ACs. A total of 240 proteins are shared between ECs and BPs, 326 between ECs and ACs, and 229 between BPs and ACs. The core proteome common to all three cell types comprises 2705 proteins. GO analysis of the molecular functions associated with the cell-type-specific proteins (Figure 7B) revealed the distinct and expected functional profiles observed previously in Figure 1 and Figure 2: for ECs, functions related to development, locomotion, and response to stimuli, consistent with pro-angiogenic processes; for BPs, cellular and biological regulation processes, reflecting their proliferative nature and regulation of the cell cycle; and for ACs, functions linked to cellular processes and localization, closely associated with lipid metabolism.

## 4. Conclusions

This study provides a unique comprehensive proteomic characterization of primary endothelial cells (ECs), brain pericytes (BPs), and astrocytes (ACs)—the three main cell types known to finely regulate vascular and metabolic function in the CNS—comparing and merging two sampling methods: whole-cell lysate (WCL) and subcellular fractionation strategies. We demonstrate that classical WCL-based DDA workflows, while effective for global protein profiling, systematically underrepresent poorly soluble and compartmentalized proteins such as those associated with membranes or the cytoskeleton [59,60,61]. In contrast, subcellular fractionation significantly enhances proteome depth, yielding a +56.7% to +92.8% increase in total protein identifications depending on the cell type.

By compiling non-redundant proteomes across cytoplasmic (F1), membrane (F2), nuclear (F3), and insoluble (F4) fractions, we defined enriched datasets (NRP Fr.) that offer improved detection of biologically relevant sub-proteomes, including a substantial gain in plasma membrane (PM) protein identification [62,63,64]. Despite this enrichment, our data also reveal that sub-proteome-specific sampling can introduce biases. For example, direct comparisons between WCL and fractionated data show discordant identification of certain PM proteins, attributable to differential recovery across fractions or loss of peripheral membrane-associated proteins during biochemical separation [60,65,66]. Moreover, although fractionation methods are valuable for improving access to cellular subproteomes, certain complex transmembrane proteins-particularly those with more than four transmembrane domains, such as ATP-binding cassette (ABC) transporters-remain poorly represented or undetected. This limitation is likely related to inefficient tryptic digestion of such highly hydrophobic proteins and can be mitigated, as demonstrated in BBB ECs using membrane-targeted strategies [67]. Thus, while fractionation approaches provide a useful overview of subproteomes, further methodological optimization is required to achieve more comprehensive coverage.

We further show that interpretation of cell-type specificity is sensitive to the sampling method. ADAM10, a pleiotropically expressed membrane protease with 60 to 100 known substrates [21,68,69,70], appeared EC-specific under WCL conditions but was broadly detected across all three cell types following fractionation. Similarly, GFAP, typically considered an astrocyte marker, was unexpectedly identified in EC samples, likely reflecting transient expression in immature endothelial populations, as previously reported during early BBB development [71,72,73,74]. These findings illustrate the critical importance of considering both protein sampling strategy and cellular differentiation status when defining cell-type-specific markers. Moreover, as illustrated in ACs, culture–medium composition is a critical parameter when interpreting cellular phenotype and differentiation state, because in vitro culture conditions may generate marker-expression profiles that do not fully recapitulate those observed in vivo.

To mitigate these limitations and provide a resource for the field, we generated integrative reference proteomes for ECs, BPs, and ACs by pooling all DDA-based identifications from both WCL and NRP Fr. strategies. These reference datasets comprise 3605 proteins for ECs, 3378 for BPs, and 3976 for ACs, with consistent sub-proteome representation (∼33% membrane proteins and ∼11.5% PM proteins), and functionally enriched categories that reflect the known biology of each cell type [75,76].

Altogether, our findings support a dual conclusion. First, that subcellular fractionation significantly improves proteome coverage and sub-proteome resolution. Second, interpretation of proteomic data, particularly with regard to cell-type specificity, must account for cell origins, i.e., primary cells or immortalized cell lines, methodological factors (number of biological replicates, digestion parameters, composition of cell culture media), and biological contexts. The integrative proteomes provided herein constitute a valuable benchmark for neurovascular proteomics and may support future efforts in biomarker discovery, cell-type classification, and systems-level modeling of the blood–brain interface.

## Figures and Tables

**Figure 1:**
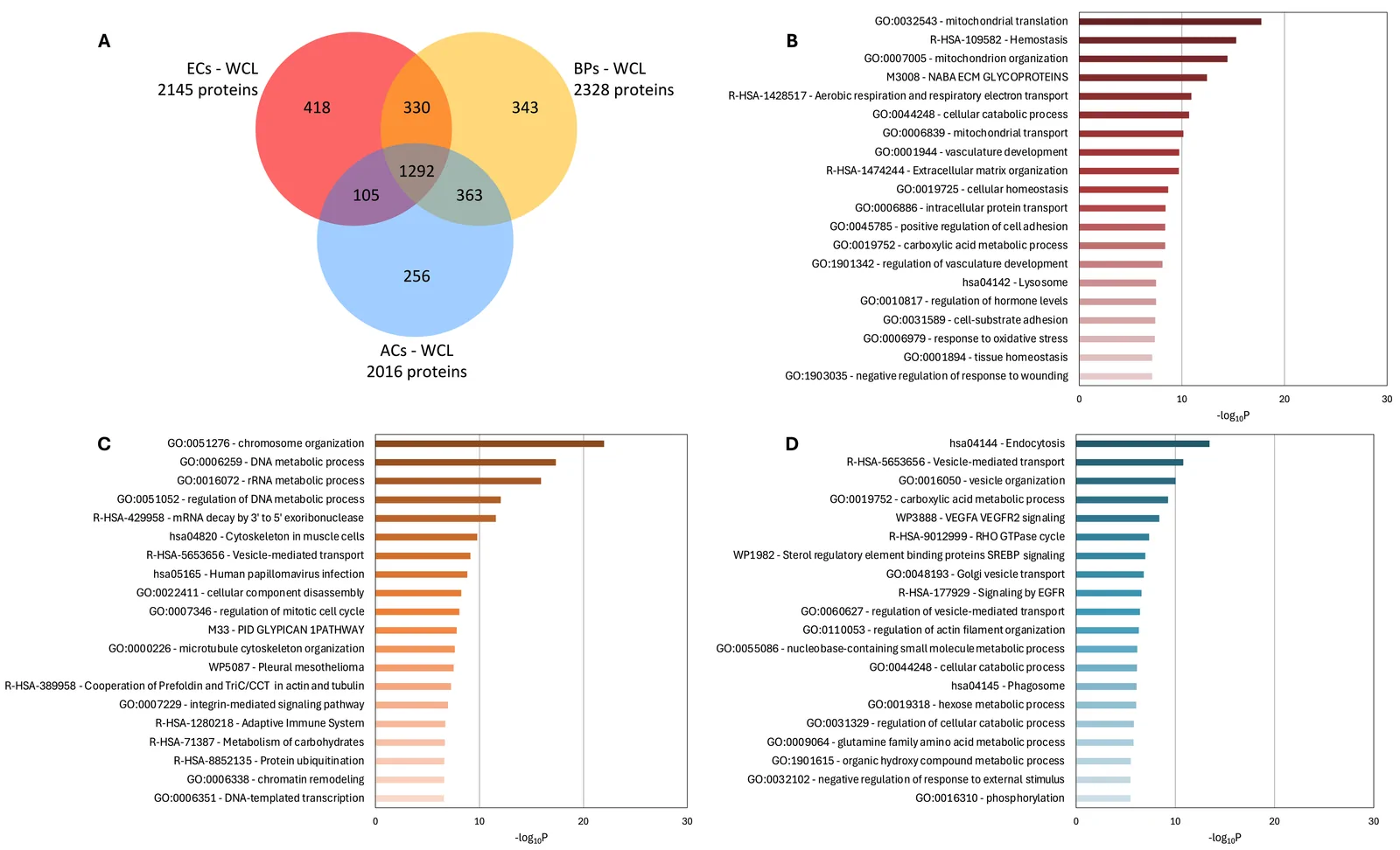
Identification of proteins in whole-cell lysates (WCL) of endothelial cells (ECs), brain pericytes (BPs), and astrocytes (ACs). (**A**) Venn diagram. (**B**–**D**) Gene Ontology (GO) analysis of biological processes: (**B**) ECs, (**C**) BPs, (**D**) ACs. Bar length and color intensity indicate enrichment significance, expressed as −log_10_*P* and calculated by Metascape using a cumulative hypergeometric test; higher values denote more significant enrichment.

**Figure 2:**
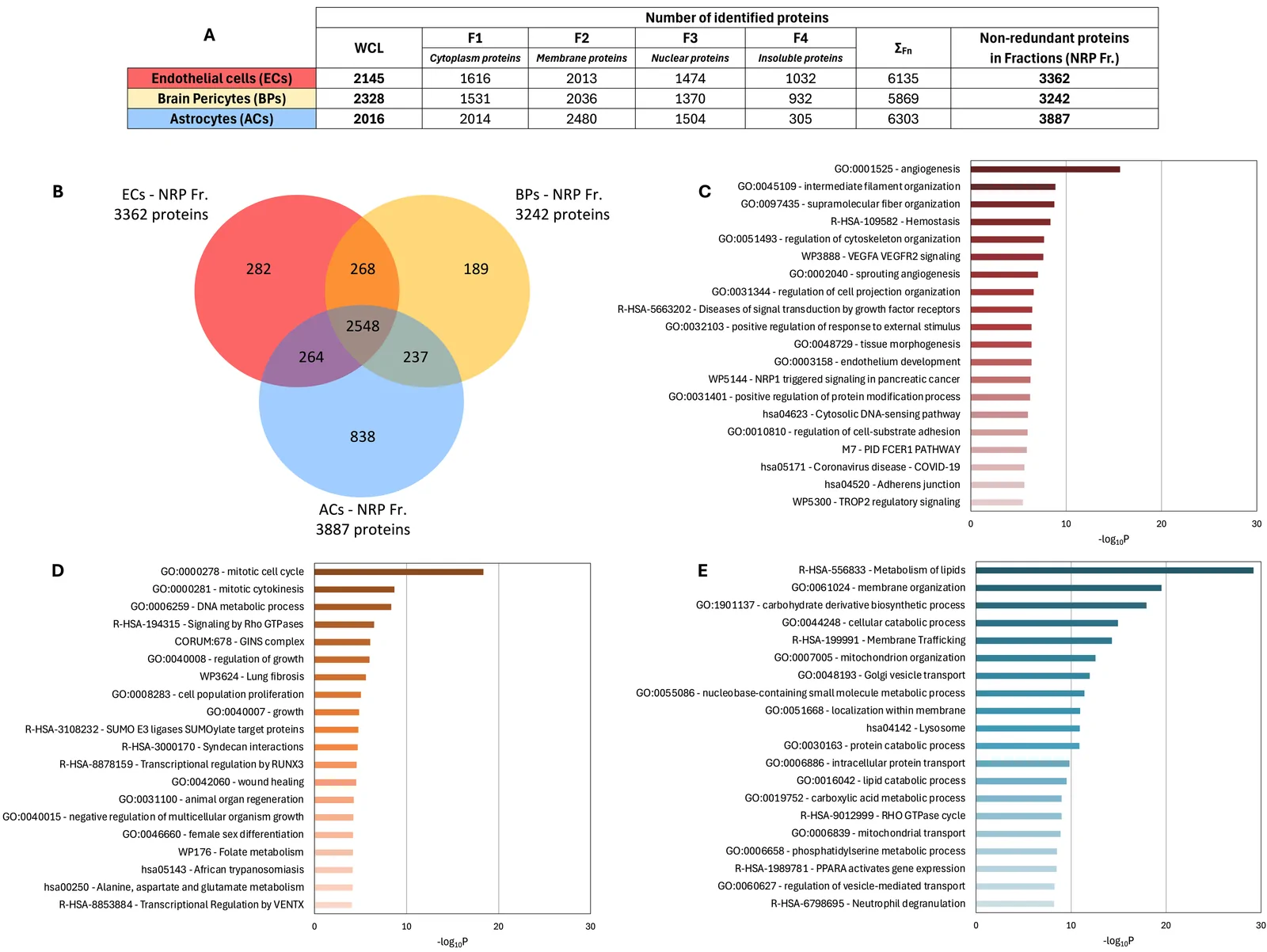
Protein identification following subcellular fractionation. (**A**) Number of proteins identified in each fraction and the corresponding non-redundant proteins (NRP Fr.) compared to WCL. (**B**) Venn diagram. F1: cytoplasmic proteins; F2: membrane proteins; F3: nuclear proteins; F4: insoluble proteins. (**C**–**E**) GO analysis of biological processes: (**C**) ECs, (**D**) BPs, (**E**) ACs. Bar colors ranging from dark to light indicate decreasing levels of protein enrichment within each biological process. Bar length and color intensity indicate enrichment significance, expressed as −log_10_*P* and calculated by Metascape using a cumulative hypergeometric test; higher values denote more significant enrichment.

**Figure 3:**
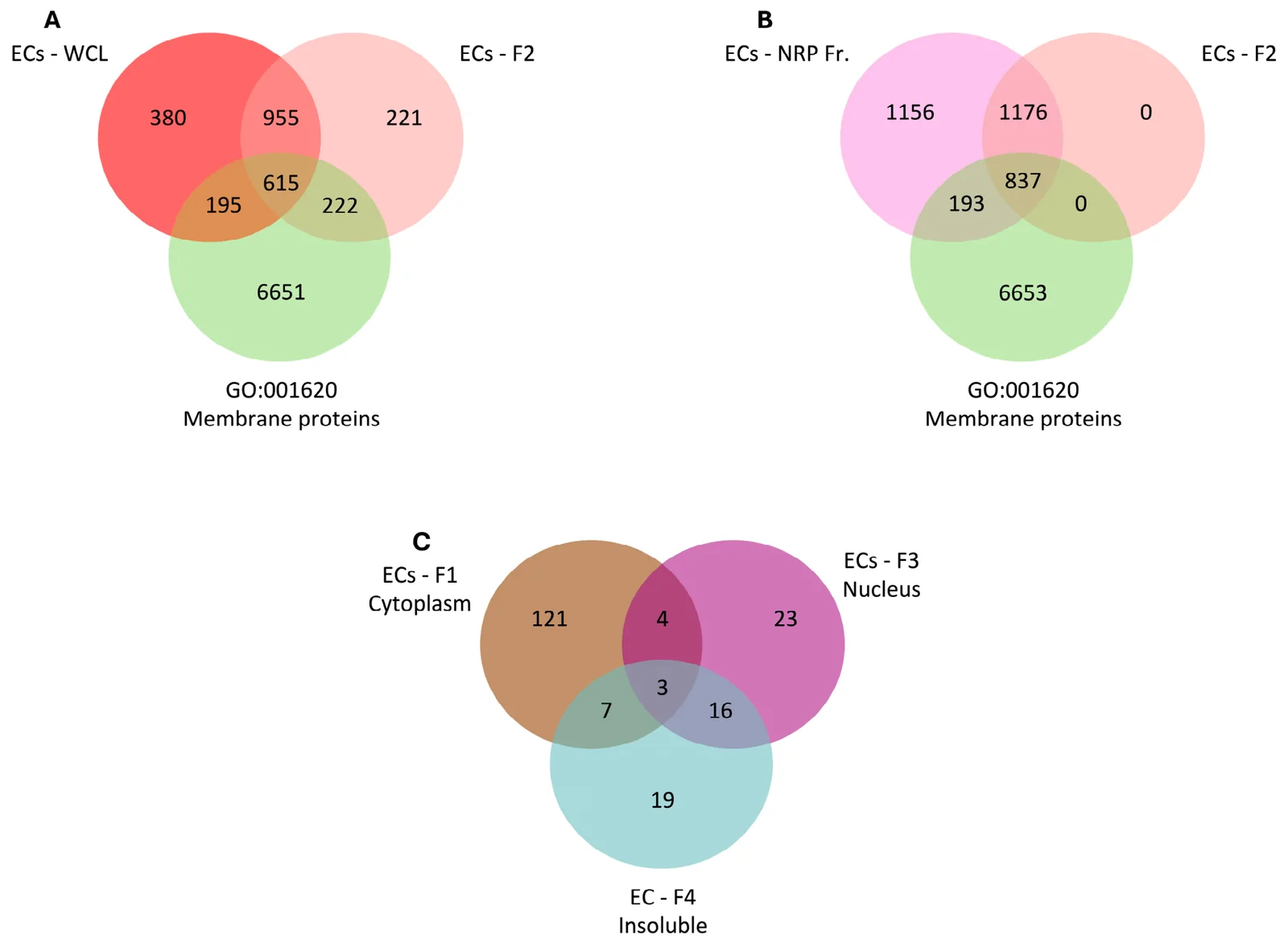
Persistence of missing membrane proteins after fractionation. (**A**) Venn diagram comparing EC membrane proteins (F2) with WCL and GO-annotated membrane proteins. (**B**) Venn diagram comparing EC membrane proteins (F2) with EC NRP Fr. and GO-annotated membrane proteins. The red circle indicates proteins absent from F2 but present in NRP Fr. (**C**) Venn diagram showing the distribution of these missing membrane proteins across other fractions.

**Figure 4:**
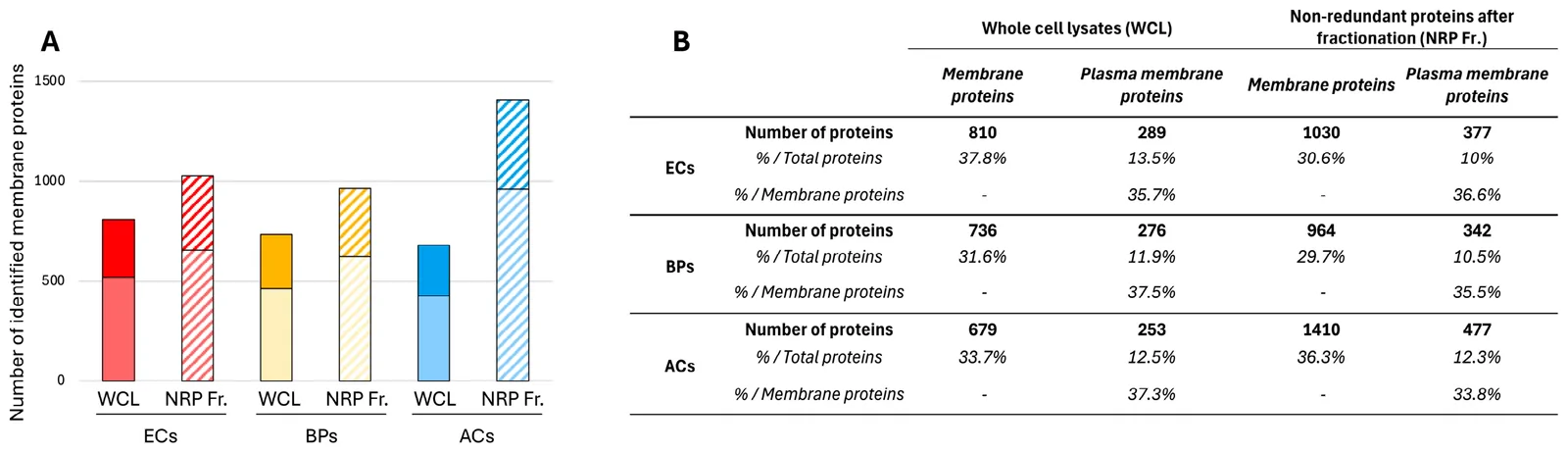
Relative enrichment of membrane and plasma membrane proteins in WCL versus NRP Fr. after fractionation. (**A**) Increase in protein identification following fractionation. (**B**) Number and relative proportion of identified proteins across ECs, BPs, and ACs.

**Figure 5:**
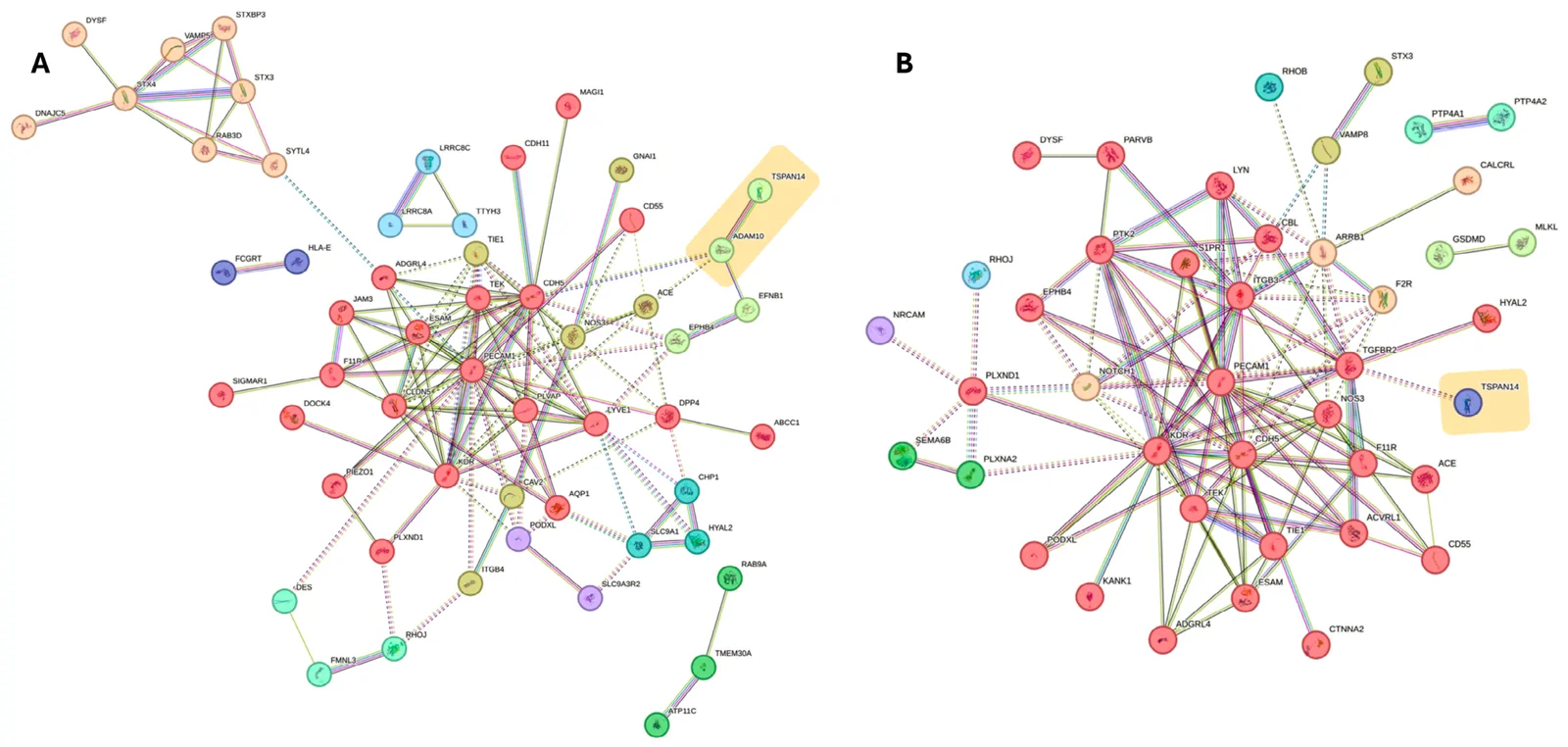
The ADAM10 case study analysis of EC plasma membrane proteins. Functional protein association networks (STRING database) from (**A**) WCL and (**B**) NRP Fr. datasets. The orange rectangle denotes the Tetraspanin-14 (TSPAN14) proxy. Edge colors denote the supporting STRING evidence channels: knowledge-based sources, assay-derived evidence, and genome-based predictions; multicolored edges indicate support from multiple sources. Only associations with a STRING combined score ≥0.400 were displayed.

**Figure 6:**
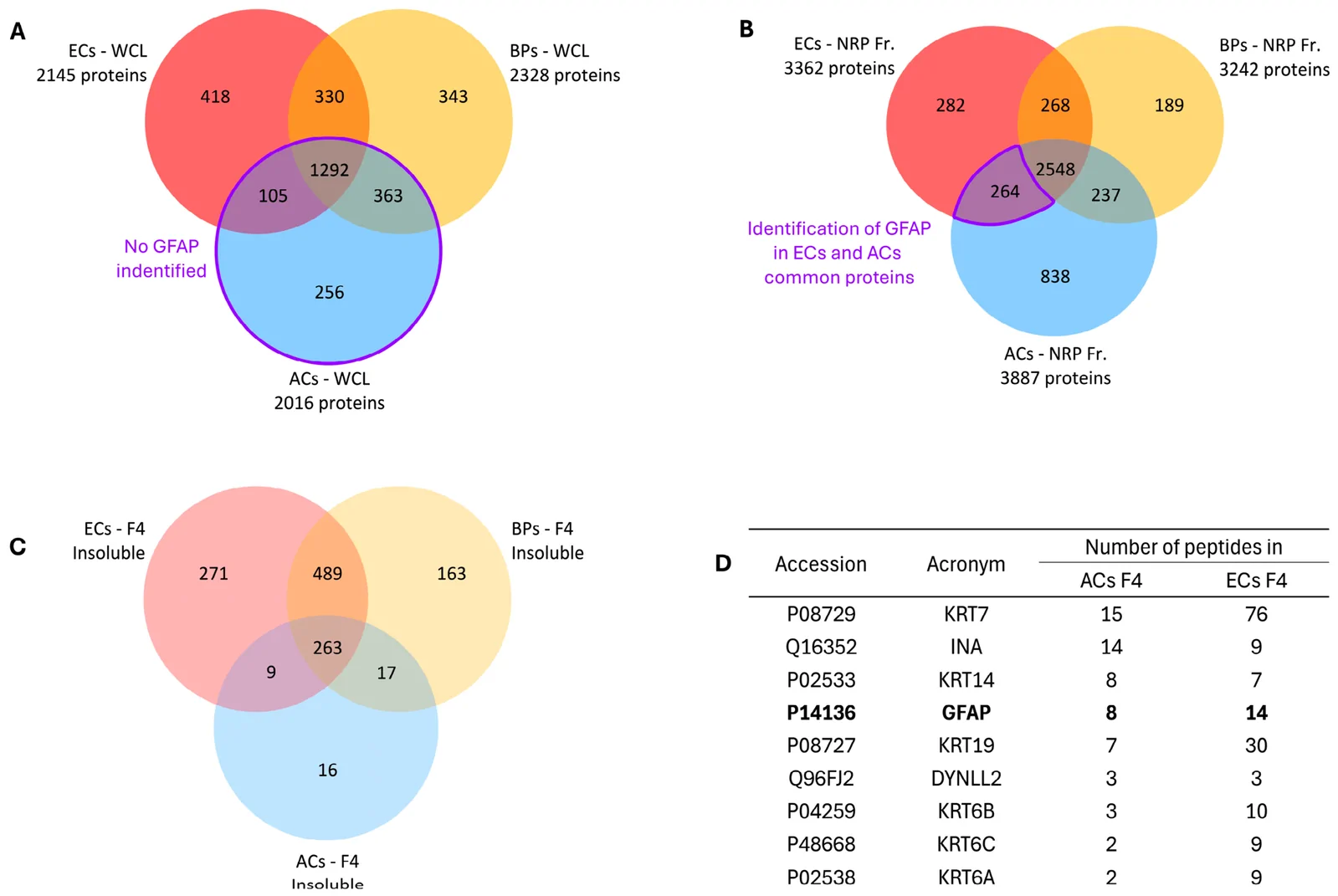
Is GFAP specific to astrocytes (ACs)? (**A**) Venn diagram comparing proteins in WCL from ECs, BPs, and ACs. (**B**) Venn diagram comparing proteins in NRP Fr. from ECs, BPs, and ACs. (**C**) Venn diagram showing the localization of these proteins in the insoluble protein fraction (F4) across the three cell types. (**D**) Table summarizing nine proteins commonly identified in the F4 fraction of both ECs and ACs.

**Figure 7:**
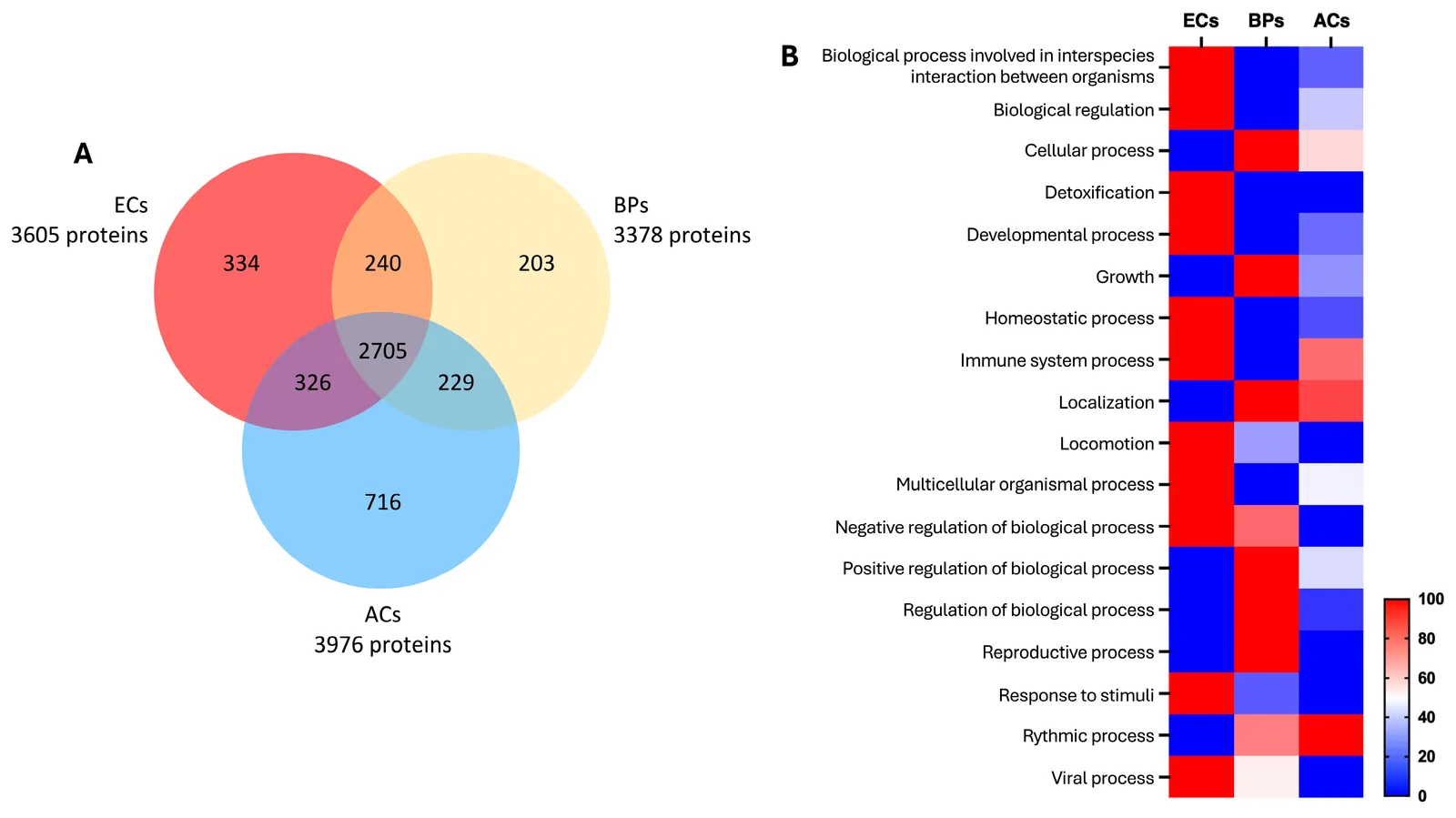
Overview of the reference proteomes of ECs, BPs, and ACs, and the main molecular functions of cell type-specific proteins. (**A**) Venn diagram. (**B**) Heatmap of major GO molecular functions. The cool-to-warm color scale indicates increasing enrichment of the identified proteins in each GO molecular function.

**Table 1:** Identified membrane proteins in whole-cell lysates (WCL), membrane protein (F2) fractions and non-redundant proteins in all fractions (NRP Fr.), and the fate of missing proteins in F2 fractions compared to NRP Fr.

Number of Identified Membrane Proteins							
	in WCL	in F2	in NRP Fr.	Missing in F2/NRP Fr.	in F1	in F3	in F4
ECs	810	837	1030	193	135	46	45
	-	103%	127%		59.7%	20.4%	19.9%
BPs	736	780	964	184	123	47	44
	-	106%	131%		57.5%	22%	20.5%
ACs	679	1191	1410	219	181	45	12
	-	175%	208%		76.1%	18.9%	5%

**Table 2:** Reference total proteomes and membrane sub-proteomes of ECs, BPs, and ACs.

		Whole-Cell Lysates (WCL)		Non-Redundant Proteins After Fractionation (NRP Fr.)	
		Membrane Proteins	Plasma Membrane Proteins	Membrane Proteins	Plasma Membrane Proteins
ACs	Number of proteins	679	253	1410	477
	%/Total proteins	33.7%	12.5%	36.3%	12.3%
	%/Membrane proteins	-	37.3%	-	33.8%
ECs	Number of proteins	810	289	1030	377
	%/Total proteins	37.8%	13.5%	30.6%	10%
	%/Membrane proteins	-	35.7%	-	36.6%
BPs	Number of proteins	736	276	964	342
	%/Total proteins	31.6%	11.9%	29.7%	10.5%
	%/Membrane proteins	-	37.5%	-	35.5%

## Data Availability

The data supporting this study are available in Supplementary Tables and from the corresponding author upon reasonable request. All the raw data are available in open access on Zenodo (https://doi.org/10.5281/zenodo.22639751, accessed on 7 September 2026).

## Supplementary Materials

The following supporting information can be downloaded at https://www.mdpi.com/article/10.3390/biology15191724/s1: Figure S1. Missing membrane proteins in BPs despite fractionation; Figure S2. Missing membrane proteins in ACs after fractionation; Figure S3. Is ADAM10 specific to ECs?; Table S1. Data-dependent acquisition (DDA)-based protein identification in WCL of ECs, BPs, and ACs; Table S2. Proteomic profiles of ECs from fractionated samples and corresponding NRP Fr; Table S3. Proteomic profiles of BPs from fractionated samples and corresponding NRP Fr; Table S4. Proteomic profiles of ACs from fractionated samples and corresponding NRP Fr; Table S5. List of identified ECs, BPs, and ACs common markers in WCL and fractionated samples; Table S6. Reference proteomes of ECs, BPs, and ACs derived from merged DDA analyses of WCL and fractionated samples.
